# A rare case of severe third degree friction burns and large Morel-Lavallee lesion of the abdominal wall

**DOI:** 10.1186/s41038-018-0108-1

**Published:** 2018-03-06

**Authors:** Darnell J. Brown, Kuo Jung G. Lu, Kristina Chang, Jennifer Levin, John T. Schulz, Jeremy Goverman

**Affiliations:** Massachusetts General Hospital, Harvard Medical School, Boston, MA USA

**Keywords:** Friction burns, Blunt trauma friction burns, Internal degloving injuries, Morel-Lavallee lesion, Traumatic pseudocyst

## Abstract

**Background:**

Morel-Lavallee lesions (MLLs) are rare internal degloving injuries typically caused by blunt traumatic injuries and most commonly occur around the hips and in association with pelvic or acetabular fractures. MLL is often overlooked in the setting of poly-trauma; therefore, clinicians must maintain a high degree of suspicion and be familiar with the management of such injuries, especially in obese poly-trauma patients.

**Case presentation:**

We present a 30-year-old female pedestrian struck by a motor vehicle who sustained multiple long bone fractures, a mesenteric hematoma, and full-thickness abdominal skin friction burn which masked a significant underlying abdominal MLL. The internal degloving caused significant devascularization of the overlying soft tissue and skin which required surgical drainage of hematoma, abdominal wall reconstruction with tangential excision, allografting, negative pressure wound therapy, and ultimately autografting.

**Conclusion:**

MLL is a rare, often overlooked, internal degloving injury. Surgeons must maintain a high index of suspicion when dealing with third degree friction burns as they may mask underlying injuries such as MLL, and a delay in diagnosis can lead to increased morbidity.

## Background

Most friction or flame burn injuries are appreciated during the primary trauma survey but may later be neglected or overlooked by more severe injuries. A friction burn injury, such as road rash, occurs when skin is abraded by contact with a hard object and often involves both physical abrasion to skin and a burn from the heat generated by the friction [1]. Morel-Lavallee lesions (MLLs) are rare traumatic injuries caused by a shearing force, which cause internal degloving of soft tissue. Other names for these lesions are post-traumatic cysts, post-traumatic pseudocysts, Morel-Lavallee effusion, or Moral-Lavallee hematoma [2–5]. Unlike friction burns to the skin, which are easy to recognize, many MLLs are often missed entirely at initial evaluation [2].

The first report of a MLL was by the French physician Maurice Morel-Lavallee, in 1853, and was associated with pelvic trauma [3, 6]. Most MLLs and closed internal degloving injuries have been described in the orthopedic literature, typically associated with traumatic injuries to the extremities, pelvic region, or greater trochanter. MLLs occur when a sheering force creates an open space between the skin and fascia which, over time, fills with blood and/or serous fluid and then has the potential for infection or organization into more chronic cystic structures. Disrupted perforating vessels along fascial planes are the main source of continued fluid accumulation, and given the large capacity for fluid in the thigh, pelvic, and abdominal regions (especially in obese patients), patients with MLL may require larger volume resuscitation and may demonstrate ongoing blood or fluid requirements. The reported incidence of MLL’s in the literature ranges from 2 to 12% in the setting of pelvic fractures [7–9]. We describe a case of a full-thickness abdominal friction burn overlying, and complicated by, an MLL internal degloving injury.

## Case presentation

**Fig. 1:**
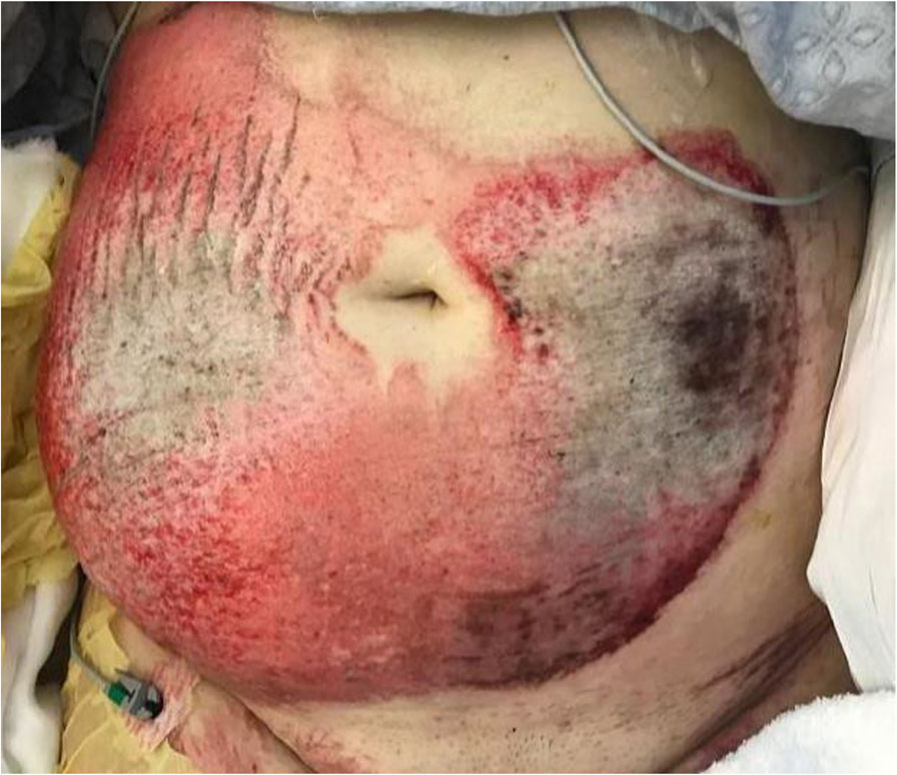
Initial evaluation of mixed friction burn to anterior abdomen of a 30-year old female pedestrain struck by a motor vehicle. Total burn surface area (TBSA) estimated to be 13% (4% full-thickness 3rd degree, 9% partial thickness 2nd degree)

**Fig. 2:**
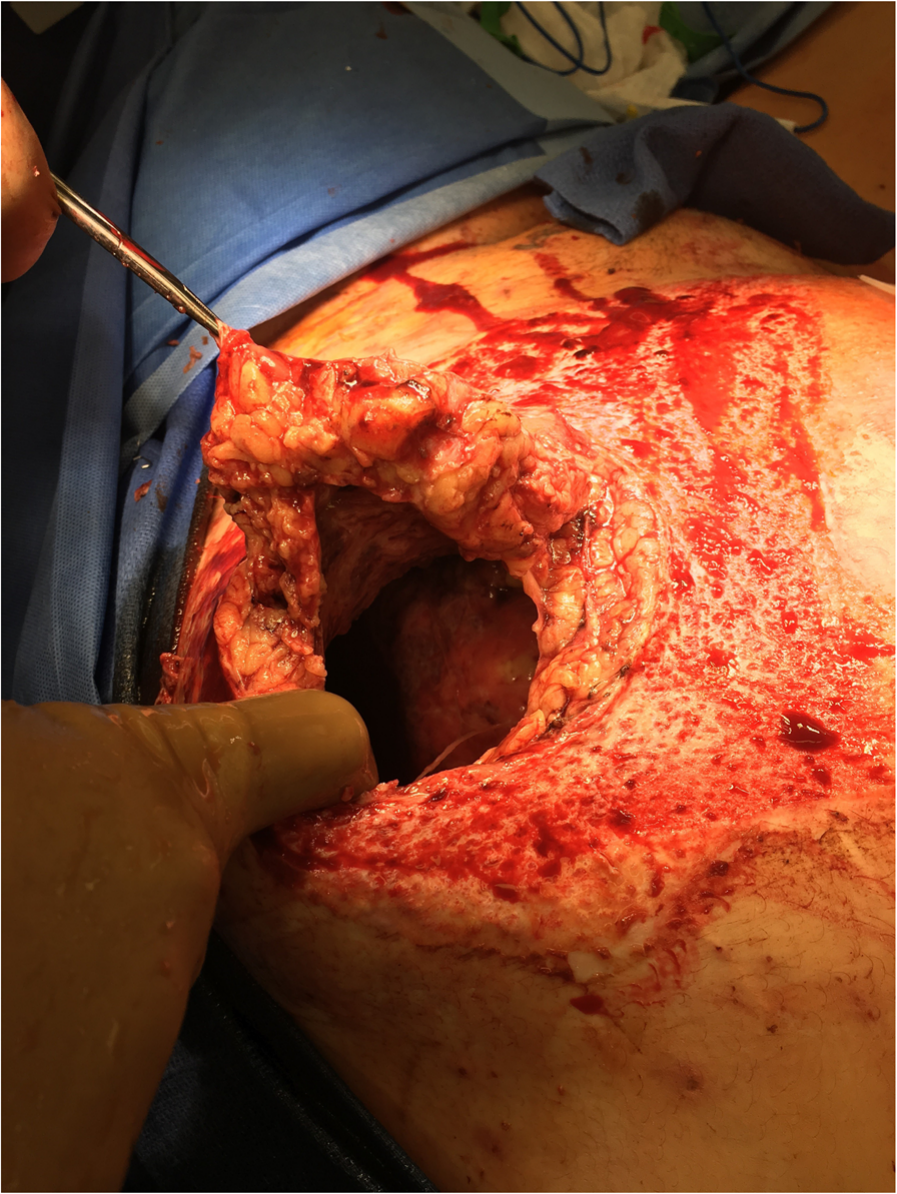
Morel-Lavallee lesion discovery during of a 30-year old female pedestrain struck by a motor vehicle tangential burn wound excision revealed full-thickness abdominal wall ischemia (approximately 12 cm diameter), extensive fascial-cutaneous separation, and traumatic dissection. Over 1.5 L of fluid were removed from this region at index operation requiring drain placement for weeks post-operation

**Fig. 3:**
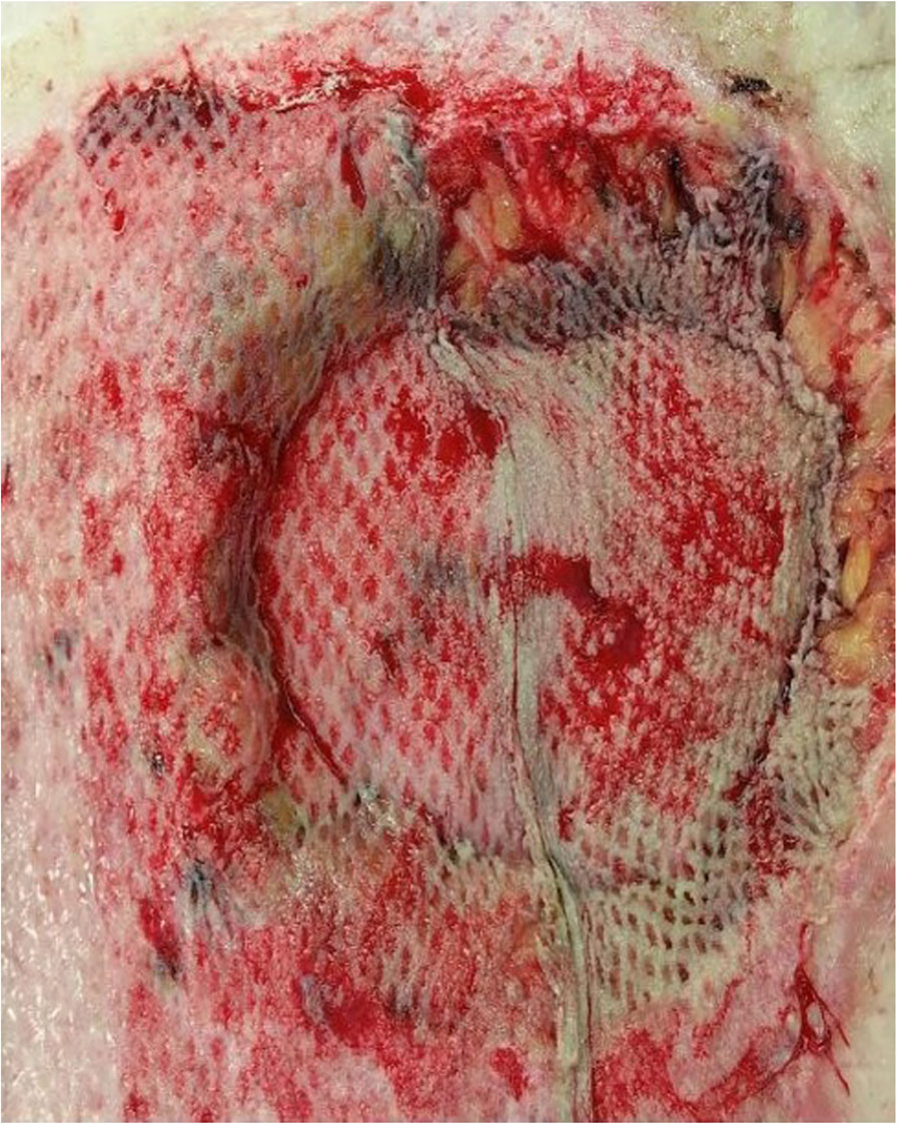
Post-repair day 5 of abdominal wall Morel-Lavallee lesion with excision and autografting with split-thickness skin graft of a 30-year old female pedestrain struck by a motor vehicle

**Fig. 4:**
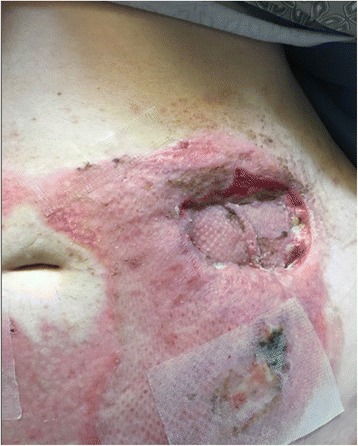
Post-repair week 5 of abdominal wall Morel-Lavallee lesion with overlying burn injury of a 30-year old female pedestrain struck by a motor vehicle. Successful take of split thickness skin graft

A 30-year-old obese (BMI 35.3) female was struck by a tow truck and dragged for 30 ft. Initial trauma evaluation revealed a left sided closed femur fracture, treated with intramedullary (IM) nailing, and a humerus fracture with open reduction internal fixation (ORIF). Additional radiologic findings included a small bowel mesenteric hematoma, managed non-operatively, a small left flank hematoma, and six-rib fractures. The burn service was consulted on hospital day 5 for management of her abdominal friction burns (TBSA 13%), of which 4% appeared to be full thickness on initial evaluation [Fig. 1]. The patient was taken to the operating room (OR) for an uncomplicated tangential excision and allografting of the abdominal friction burn. Upon return to the OR 5 days later for planned excision of allograft and placement of autograft, significant progression of burn depth and adipose necrosis was noted and the left flank was distended and edematous. Upon further excision of necrotic adipose tissue, the underlying fascial-cutaneous separation (MLL) was discovered, and approximately 1.5 L of serosanguinous fluid was evacuated from this cavity [Fig. 2]. To prevent re-accumulation of fluid into this dead space, a 19F round Blake drain was placed in the dependent portion of the MLL, the overlying cutaneous defect was grafted with a 2:1 meshed split-thickness skin autograft, a negative pressure dressing was used to bolster the graft, and an abdominal binder was worn at all times. During the first 24 and 48 post-operative hours, 700 and 1055 mL of serosanginous fluid were drained, respectively. The primary dressings were removed 5 days post-autografting with satisfactory results [Fig. 3]; after 5 weeks, autograft take was noted to be > 95% with further aesthetic improvements appreciated [Fig. 4].

## Discussion

Friction burns are often the result of high-energy trauma, and therefore, the associated injuries typically take precedence with respect to the timing of treatment and triage. This delay in treatment can result in additional morbidity. While the etiologies of deep friction burns in children are commonly treadmills [10, 11] and vacuum cleaner belts [11], deep friction burns in adults are most often associated with high-energy trauma and, as such, with long bone fractures, pelvic fractures, and/or head injuries [12]. The majority of burns caused by friction are superficial; however, pedestrians struck by motor vehicles on high-temperature road surfaces are more likely to sustain third degree friction burns and therefore require excision and skin grafting. Since the initial insult to the skin is a thermal burn injury caused by friction, the zone of injury may not be defined at the acute evaluation and may need observation for declaration of ischemia. If the skin and/or subcutaneous tissues are traumatically separated from the overlying fascia (MLL), as in the case presented here, perfusion to the overlying skin is further compromised from transection or thrombosis of perforators, thus creating a more complex soft tissue injury.

Obesity also complicates the discovery of MLL and may contribute to delays in diagnosis, as was the case for the patient presented here. Furthermore, research suggests that obesity and diabetes are associated with abnormal dermal functioning, and obese murine models suggest a reduction in dermal layer favoring more adipose tissue, particularly in the deep dermis (hypodermis) as well as in the subcutaneous adipose tissue, which potentially allows the skin to slide easier over the deep fascia of the trunk [6].

A degloving injury is a separation of the skin and subcutaneous tissue from the fixed underlying fascia, which can compromise capillary perfusion and blood vessels traveling through this island of tissue. MLLs are a form of closed degloving injury caused by trauma that delivers a shearing force to the soft tissues [6]. MLL occurs most commonly around the hip and is well described in the orthopedic literature. These lesions require early surgical intervention to prevent complications from seroma and hematoma formation and subsequent infection of these collections. Surgical intervention consists of incision and drainage or, in more severe cases, debridement of overlying devascularized soft tissue. In the acute setting, computerized tomography (CT) scanning may demonstrate a small, simple hematoma, as it may take some time for the potential space to fill. Active arterial extravasation is noted in less than 1/3 of cases [13]; therefore, the evolution and continued drainage of hemolymphatic fluid increases radiologic accuracy over time. The average measurement of Hounsfield units (HU) reflect the internal contents of MLLs varying based on age: acute 30 HU, subacute 16 HU, and chronic 6 HU [14].

The natural history of MLL has yet to be completely described [14] but are typically classified into three different subtypes based on imaging: seroma, subacute hematoma, and chronic hematoma. The genesis of the fluid accumulation is typically from disruption of blood vessels and lymphatics in the subcutaneous space overlying the fascia of the muscle. Chronic fluid accumulation can then become infected and turn into an acute abscess or encapsulated by a fibrous capsule over time if the collection remains sterile. Ultimately, the discovery and diagnosis of MLL is based on several factors, including skin mobility, subcutaneous fluctuation, decreased cutaneous sensation, tire marks, and friction burns [14].

The treatment options for MLL are based on the clinical presentation and timing of diagnosis. Patients with MLL after trauma always have compromised circulation to the skin and subcutaneous tissue in the injured segment and it is often difficult to determine the extent of injury and long term viability of the overlying tissue [14]. Treatment options include serial excisional debridement followed by healing by secondary intention or skin grafting, percutaneous drainage, or sclerotherapy. There are no set standards or guidelines for the management of such lesions although the Mayo Clinic experience suggests operative intervention is required when more than 50mL of fluid has been aspirated from a patient being treated conservatively [14].

## Conclusions

Surgical drainage is required for the management of large MLLs. Autograft application to replace devitalized tissue offers the most predictable and desired results. Negative pressure therapy aids in resolution and prevention of fluid re-accumulation. Patients become symptomatic as fluid accumulates which prompts detection. Secondary infection of the MLL may present with fevers, leukocytosis, cellulitis, or pain. Our case is unusual because it did not occur over a fixed prominent bony structure and complicated by full thickness friction burn. Providers must maintain a high degree of clinical suspicion when managing deep dermal abrasions in fluctuant areas in morbidly obese polytrauma.

## Abbreviations

CT: Computerized tomography
HU: Hounsfield Units
MLL: Morel-Lavallee lesion
STSG: Split-thickness skin graft
TBSA: Total body surface area
